# The impact of livestock on the abundance, resting behaviour and sporozoite rate of malaria vectors in southern Tanzania

**DOI:** 10.1186/s12936-014-0536-8

**Published:** 2015-01-21

**Authors:** Valeriana S Mayagaya, Gamba Nkwengulila, Issa N Lyimo, Japheti Kihonda, Hassan Mtambala, Hassan Ngonyani, Tanya L Russell, Heather M Ferguson

**Affiliations:** Environmental Health and Ecological Sciences, Ifakara Health Institute, PO Box 53, Ifakara, Tanzania; Department of Zoology and Wildlife Conservation, University of Dar es Salaam, PO Box 35065, Dar es Salaam, Tanzania; Faculty of Medicine, Health and Molecular Sciences, James Cook University, PO Box 6811, Cairns, Queensland 4870 Australia; Boyd Orr Centre for Population and Ecosystem Health, Institute of Biodiversity, Animal Health and Comparative Medicine, University of Glasgow, G12 8QQ Glasgow, UK

**Keywords:** Malaria transmission, Environmental management, Vector behaviour, Ecology, Zooprophylaxis, Kilombero Valley

## Abstract

**Background:**

Increases in the coverage of long-lasting insecticidal nets (LLINs) have significantly reduced the abundance of *Anopheles gambiae sensu stricto* in several African settings, leaving its more zoophagic sibling species *Anopheles arabiensis* as the primary vector. This study investigated the impact of livestock ownership at the household level on the ecology and malaria infection rate of vectors in an area of Tanzania where *An. arabiensis* accounts for most malaria transmission.

**Methods:**

Mosquito vectors were collected resting inside houses, animal sheds and in outdoor resting boxes at households with and without livestock over three years in ten villages of the Kilombero Valley, Tanzania. Additionally, the abundance and sporozoite rate of vectors attempting to bite indoors at these households was assessed as an index of malaria exposure.

**Results:**

The mean abundance of *An. gambiae s.l.* biting indoors was similar at houses with and without livestock. In all years but one, the relative proportion of *An. arabiensis* within the *An. gambiae s.l.* species complex was higher at households with livestock. Livestock presence had a significant impact on malaria vector feeding and resting behaviour. *Anopheles arabiensis* were generally found resting in cattle sheds where livestock were present, and inside houses when absent. Correspondingly, the human blood index of *An. arabiensis* and *An. funestus s.l.* was significant reduced at households with livestock, whereas that of *An. gambiae s.s*. was unaffected.

Whilst there was some evidence that sporozoite rates within the indoor-biting *An. gambiae s.l* population was significantly reduced at households with livestock, the significance of this effect varied depending on how background spatial variation was accounted for.

**Conclusions:**

These results confirm that the presence of cattle at the household level can significantly alter the local species composition, feeding and resting behaviour of malaria vectors. However, the net impact of this livestock-associated variation in mosquito ecology on malaria exposure risk was unclear. Further investigation is required to distinguish whether the apparently lower sporozoite rates observed in *An. gambiae s.l*. at households with livestock is really a direct effect of cattle presence, or an indirect consequence of reduced risk within areas where livestock keepers choose to live.

**Electronic supplementary material:**

The online version of this article (doi:10.1186/s12936-014-0536-8) contains supplementary material, which is available to authorized users.

## Background

The increasing use of intradomiciliary-based control measures such as long-lasting insecticide-treated nets (LLINs) and indoor residual spraying (IRS) has shown substantial success in reducing malaria transmission in sub-Saharan Africa [1-3]. The success of LLINs and IRS is mainly due to their effective targeting of indoor-biting, highly anthropophilic vectors such as *Anopheles gambiae s.s.* [1,4,5]. However these methods are less effective at controlling vectors that bite at dusk, rest outside the home (exophilic) and feed on livestock (e.g., zoophagic) as well as humans [6-11]. Recently, the abundance of highly anthropophilic, endophilic vector species such as *An. gambiae s.s.* has declined relative to more behaviourally plastic species such as *Anopheles arabiensis* in areas of high LLIN coverage [7,12-14]. Unlike *An. gambiae s.s.*, *An. arabiensis* will readily feed and rest outside as well as inside houses, and feed on cattle [15-21]. At present, few outdoor-based control measures exist to effectively target this and other vector species with exophagic behaviour. Several potential methods for controlling outdoor-biting mosquitoes are under development (e.g., outdoor-based, vector-killing stations [22,23], biological control [24] and use of insecticide-treated livestock [25-27]), but at present there is no standard method under routine operational use. The successful implementation of all these methods would benefit from clear understanding of the ecology and behaviour of vectors outside of domestic environments [28].

The potential use of alternative host species to divert malaria vectors away from people has long been recognized as a potential environmental strategy for the reduction of malaria transmission [29]. This strategy, known as zooprophylaxis, is credited with playing a major role in the elimination of malaria from Europe and other temperate areas following an increase in livestock keeping [30]. However, increasing the availability of alternative hosts such as livestock could alternatively enhance human malaria exposure (e.g. “zoopotentiation”) if the heat and odour cues emitted by animals attract a greater number of vectors to households in or near where they are kept [31]. Also zoopotentiation could occur if the physical disturbances created by animals (e.g., puddles, hoof prints, watering sites) increases larval habitat [32] and thus adult vector density near households. There have been relatively few investigations of the impacts of household cattle ownership on malaria exposure rates in Africa, and their results have been mixed. Whereas some studies have reported an association between livestock keeping and reduced mosquito biting rates and malaria risk [31,33,34], others have found no effect [21,35]. In the latter case, the study was conducted in a setting where the dominant vector species was highly anthropophilic and endophilic (*An. gambiae s.s.*) [21,35]. This may account for the absence of any zooprophylactic effect in contrast to settings where *An. arabiensis* is prevalent [33,34]. Further investigation of zooprophylaxis within rapidly transmission settings dominated by zoophilic, exophilic vectors is thus needed to fully assess the potential of this approach.

In Tanzania, malaria is endemic in many parts of the country and is the leading public health problem [36,37]. The Kilombero Valley in south-eastern Tanzania experiences year-round malaria transmission due to the presence of *An. arabiensis*, *An. gambiae s.s*. and *Anopheles funestus* [38]. Livestock keeping within the region increased significantly over the past decade due to the immigration of pastoralists from other parts of the country (i.e. from 42,385 to 55,994 cattle in 2001–03 livestock census, (DALDO Kilombero district livestock department (2003), Brehony *et al.* unpublished reports), The population of livestock kept increasing even after 2003 census, this was reflected by the increase in needs of health services to livestock in the Kilombero Valley time after time (district livestock officer, personal communication ). In parallel with these changes, the coverage of LLINs has significantly increased, 2004 had a coverage of 75% of untreated nets and 2009 with the coverage of 47% of ITNs [12]. Concurrent with these changes the abundance of *An. gambiae s.s.* has rapidly declined [12], with *An. arabiensis* now being responsible for the remaining transmission. The relative frequency of *An. arabiensis* with the *An. gambiae* species complex grew from 13% in 2005 [39], to 98% in 2009 [40]. The presence of this zoophilic vector in addition to smaller populations of the more anthropophilic vectors *An. gambiae s.s.* and *An. funestus* make the Kilombero Valley an ideal location to investigate the potential impact of livestock on malaria vector ecology and human exposure risk. A three-year field study was conducted here to estimate the impact of local household livestock ownership on: (1) the abundance and diversity of mosquito vectors, (2) the feeding and resting behaviour of vectors and finally (3) net malaria exposure risk to humans. Malaria exposure risk as estimated in terms of the total number of malaria-infected mosquito bites (*An. gambiae s.l.* and *An. funestus s.l.*) expected to be received by people sleeping indoors at night. It was hypothesized that the presence of cattle at a household could reduce human malaria exposure rates if associated with a significant change in vector behaviour towards increased feeding on cattle and outdoor resting.

## Methods

### Study area

**Figure 1:**
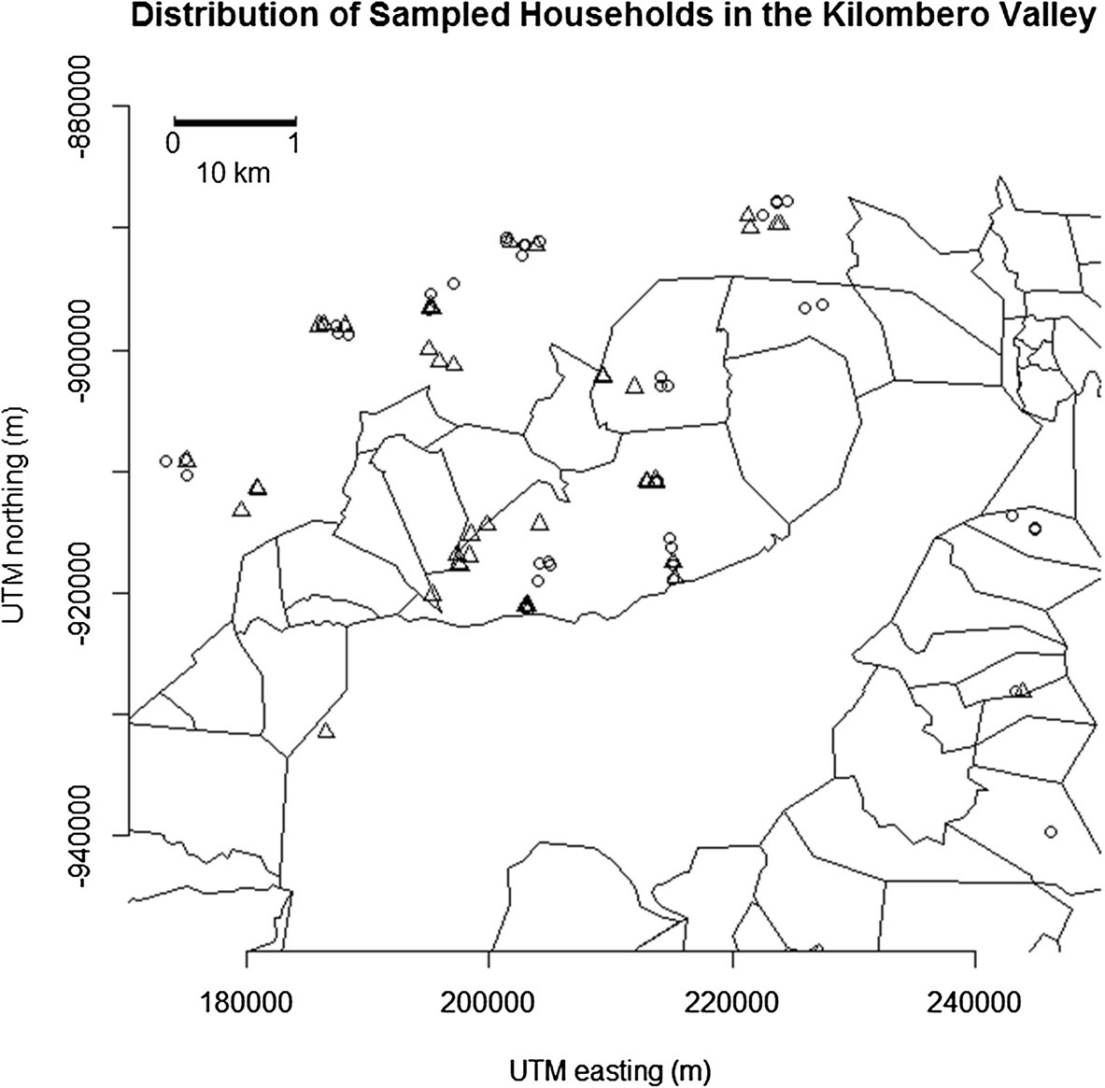
**Location of sampled households across ten villages in the Kilombero Valley.** Circles represent households where livestock were present, and triangles are households without livestock.

The study was carried out in ten villages of the Kilombero Valley (7°44’-9°26° S/35°33’-36° 56E) in the dry season of 2007 (July-October), and wet seasons (January-June) of 2008 and 2009 (March-May). All ten villages were within the area where the demographic surveillance system (DSS) of the Ifakara Health Institute (IHI) [41] has been collecting health and economic information from approximately 25,000 households each year [41]. Information from the DSS for the year preceding this study (2006) indicated that the percentage of households that owned cattle varied from less than 1 to over 16% across the ten study villages (Table 1). Longitudinal sampling was conducted at households in this region as described below (Figure 1).

**Table 1:** **Reported rates of household-level cattle ownership, associated ‘cattle availability’ strata (low, intermediate and high) and the proportion of households reporting ownership of bednets, treated and untreated, just prior to the start of this study in 10 study sites**

**Village**	**Cattle strata**	**Total households surveyed**	**% of cattle ownership**	**% of households with bed nets**	**% of households with treated bed nets**
Idunda	Low	361	4.71	0.97	0.60
Lupiro	Low	403	2.82	0.91	0.43
Mbingu	Low	1315	0.08	0.92	0.41
Idete	Intermediate	1057	7.38	0.88	0.61
Minepa	Intermediate	516	6.59	0.91	0.29
Namawala	Intermediate	955	5.45	0.92	0.65
Iragua	High	757	11.62	0.88	0.63
Kidugalo	High	524	16.79	0.93	0.64
Mkangawalo	High	1059	9.73	0.89	0.62
Sagamaganga	High	546	11.72	0.95	0.63

Information was collected by the IHI Demographic Surveillance System approximately 6 months before the start of this study (2006).

### Household selection

Census lists of households with and without livestock were obtained for the ten study villages based on the 2006 IHI DSS data. Starting in 2007, four households that reported to own livestock, and four that did not, were randomly selected for each village in the high and intermediate cattle strata groups. In villages reporting no or very low rates of cattle ownership (low cattle stratum, Table 1), only four households without livestock were initially selected (total of 68 households in year 1). In the second year (2008), livestock keeping increased in all study villages, including those in the low cattle strata, this was due immigration of new pastoralists in some villages (IHI-DSS 2008 data, unpublished data) and in others was after the introduction of a project that hired pregnant dairy cattle to a family with a purpose of improving the family’s wealth condition and they were supposed to return a calf and continue to stay with the mother. (Ulanga district village officers, personal communication). Thus it was possible to recruit an additional four households with livestock for villages in the low cattle stratum, so that a total of eight households (four with cattle, four without) were surveyed in all villages (80 households in total). A similar programme of sampling was conducted in 2009 with two changes: (1) the number of households sampled per village was reduced from eight to six (three with cattle, three without) to accommodate the long travel time to go between selected households each day and (2) two sites were dropped from the study because one became inaccessible due to heavy rains (Mkangawalo), and mosquito vector densities at another were too low for analysis (Namawala). Whenever possible, the originally selected households in each village were repeatedly sampled in all study years, but some had to be replaced due to people moving away or withdrawing consent for further collections.

### Mosquito collections

In each year, mosquito collections were conducted over a one to two month period in which each village was visited sequentially. Four continuous days of sampling were conducted in each village, with visits between villages separated by two to three days. Collections were made from all selected houses using each of the follow methods: (1) outdoor resting catches, (2) indoor resting catches, (3) resting catches in animal sheds (at houses with livestock) and (4) using CDC light traps indoors. All trapping methods were conducted daily at each household over the 4-day sampling period. Additional file 1A shows an example of typical house of most livestock keepers in Kilombero Valley.

Outdoor resting collections were conducted using artificial resting boxes made from cardboard boxes (43 × 43 × 26 cm) that had their inside lined with a black cloth [42]. Four to eight resting boxes were set each night per household. Boxes were randomly placed outside but within 5 m from houses and cattle sheds (where present). The evening before collection, resting boxes were set lying on their side, with the side closest to the nearest structure (house or cattle shed) being left open (Additional file 1C). All resting boxes were checked within the early morning hours (06.00-09.00 am), and mosquitoes resting inside them collected by aspiration (Additional file 1D). A wooden stick was placed vertically inside each box to maintain its open, square structure when in use. Boxes generally stayed intact over the sampling night, but were occasionally replaced by new boxes when some deterioration of structure was observed (e.g. due to cardboard becoming wet due to rainfall over night).

Resting catches inside houses were conducted by aspiration (2007: mouth aspirator, 2008–9: CDC backpack aspirator). During these collections, two people searched inside a house for approximately ten minutes (scanning all walls and roofs). At households where livestock were present, additional resting collections were made inside cattle sheds. In the Kilombero Valley, adult cattle are usually kept outside within a fence that is sometimes covered by a thatch roof, and calves and smaller livestock are kept within enclosed sheds with thatch roofs and walls. These cattle sheds are usually situated close to houses (Additional file 1B) (e.g. ≤ 25 m, personal observation). Mosquito resting catches were conducted in cattle sheds (from the roof and walls) in a similar way as to inside houses.

Collection of mosquitoes attempting to feed on people indoors was conducted using a CDC light trap placed in the main sleeping room of the house [43]. The trap was suspended at approximately 1.5 m above the floor and adjacent to the foot of a bed containing sleepers who were protected by an existing bed net. Traps were run between 19.00 pm and 06.00 am hours each night. The sporozoite rate of mosquitoes collected in CDC light traps was estimated as a measure of human exposure to infectious mosquito bites.

Mosquitoes captured by all trapping methods were killed by asphyxiation with chloroform. Those morphologically identified as belonging to the *An. gambiae s.l.* species complex, and *An. funestus s.l.* were preserved in 1.5 ml Eppendorf tubes containing desiccant crystal and taken to the IHI laboratory for further laboratory analyses as described below.

### Laboratory analysis

PCR analysis was conducted on a subsample of approximately 24% of all *An. gambiae s.l.* mosquitoes caught in CDC light traps (n = 22,035, Additional file 2) and resting collections (n = 4,771), respectively, to identify them to species level. PCR analysis was not conducted on *An. funestus s.l.* as they are less abundant and were considered of secondary importance. Sporozoite ELISA was performed on 57% of the collected host seeking vectors Blood meal identification by ELISA was also performed on all individually identified, blood-fed *An. gambiae s.l.* caught in resting collections [44]*.* Analyses were conducted to test for the presence of human, bovine, dog, goat, or chicken blood in the mid-guts/abdomens of blood fed mosquitoes. Initially blood meal identification analysis was conducted only on *An. gambiae s.l.* samples (2007), but in 2008 and 2009 *An. funestus s.l.* samples were also included.

A subsample of 29–99.8% (per year) of female *An. gambiae s.l.* collected in CDC light traps were individually tested for sporozoite infection by ELISA [45,46] (Supplementary Information 1). These females were also individually analysed by PCR for species identification. In 2008 and 2009, sporozoite analysis was also conducted on an additional subset of *An. gambiae s.l.* that had not been individually identified to species by PCR. While analysis of pooled *An. gambiae s.l.* compromised our ability to identify the impacts of cattle on sporozoite rates in *An. arabiensis* and *An. gambiae s.s.* separately, doing so was appropriate for addressing our main aim of estimating whether the total number of infected mosquito bites expected to be received (irrespective of vector species) was related to livestock presence. Furthermore, substantially larger numbers of mosquitoes could be tested for sporozoites when analysed in pools of *An. gambiae s.l.* rather than on individual mosquitoes. Given that sporozoite rates are often <1% , sample sizes of several thousand mosquitoes are required to achieve sufficient statistical power to robustly test for variation in infection rates between treatments. Pooling of *An. gambiae s.l.* samples for analysis of sporozoites allowed these sample size requirements to be met. Groups of *An. gambiae s.l.* were tested for the presence of sporozoites in pools of five. As sporozoite infection rates for all three vector species within the Kilombero Valley are typically less than 2% [47], it was assumed any mosquito pool that tested positive for sporozoites was the result of only one mosquito within it being infected. A subsample of female *An. funestus s.l.* caught in light traps in 2008 and 2009 were also tested for sporozoites using the ELISA method (Additional file 2).

### Statistical analysis

Variation in the daily abundance of mosquito vectors caught in CDC light traps and resting collections was analysed using generalized linear mixed models in the R statistical software package [48]. As mosquito densities from all trap types were highly over dispersed, data were modeled on the basis of a negative binomial distribution using the glmmADMB package [49]. Here, the presence of livestock at a household was treated as a fixed effect, and village, household ID, and date were fit as random effects. Separate analyses were conducted for *An. gambiae s.l.* and *An. funestus s.l.*

Only a subset of mosquitoes was subjected to further molecular analysis for identification of species (within *An. gambiae s.l*.), blood meals and malaria sporozoite presence. These variables were defined and analysed as binary outcomes as follows: i) species complex: *An. arabiensis* or *An. gambiae s.s*.; ii) human blood index: human or non-human blood meal (from specimens whose blood meal could be identified); and, iii) sporozoite infection rate: infected or uninfected. Generalized linear mixed models with a binomial link function (glmer package) in the R statistical software were used to model variation in these traits. For investigation of species composition and human blood index, separate analyses were done for each study year. Here household livestock ownership was treated as a fixed effect, and village and household ID as a random effect. Due to the relatively small number of blood-fed samples available for some resting microhabitats in some years, data on the human blood index were pooled over all years for analysis.

Whilst vector abundances and behavioural traits (e.g., host choice and resting behaviour) exhibit substantial heterogeneity between households over small distances [50-53], and even between microhabitats within households (e.g., indoor *versus* outdoor [54,55], malaria transmission rates are products of human and vector population processes and thus less variable over small scales [50,56]. In the study area, households with cattle and those without were mixed heterogeneously in some villages, but in other areas there was some spatial clustering of cattle-keepers at the subvillage level. To control for any bias in estimating the impact of livestock presence on mosquito sporozoite rates that could arise due to larger-scale spatial clustering of livestock keepers, the pair-wise distance between all households was calculated and used to estimate the ‘minimum distance to nearest household with livestock’ for each location. This variable was included in analysis as a proxy for the likelihood of a household being situated in a cluster of cattle-keeping (low values) or non-cattle keeping (high values) households. Variation in sporozoite rates between households with and without livestock was thus tested using generalized linear mixed models in which livestock ownership, year and minimum distance to nearest other household with livestock were incorporated as fixed effects, and ‘household ID’ included as random effects. This analysis was conducted only on data from pools of undifferentiated *An. gambiae s.l.* as sample sizes of individually PCR-identified *An. gambiae s.l.* and *An. funestus s.l.* were not sufficiently large for robust analysis (>1,000 s required as sporozoite rates are typically <2%). However, sporozoite rates were estimated for the subsample of *An. gambiae s.l.* whose species was confirmed by PCR.

### Ethics

After identifying potential households for mosquito collection, household owners were contacted to request their participation. The purpose and nature of the study was explained to them, and those who agreed to participate provided written informed consent. If the household owner declined to participate, the participation of the next household owner on the randomly selected list was requested until the minimum quota of households per village was reached. Ethical approval for the study was obtained from the IHI Institutional Review Board (IHRDC/IRB/No.A015) and the Medical Research Coordination Committee of the National Institute for Medical Research (NIMR 1HQ/R.8a/Vol.IX/708).

## Results

### Livestock and mosquito vector abundance and diversity

Over all three years, a total of 26,806 *An. gambiae s.l.* (22,035 host-seeking indoors, 4,771 resting) and 2,587 *An. funestus s.l.* (1,639 host-seeking indoors, 948 resting) were collected.

The abundance of indoor biting *An. gambiae s.l.* was substantially higher in the wet seasons of 2008 and 2009 (5–33 per night, Table 2) than in the dry season of 2007 (<one per night, Table 2), but there was no significant difference in between households with and without livestock in any year (Table 3). *Anopheles funestus s.l.* constituted only 4.8% of the indoor biting vector population and was generally low in abundance (<two per night). The abundance of *An. funestus s.l.* followed the same pattern as *An. gambiae s.l*. of being substantially lower in the dry season of 2007 than wet seasons of 2008 and 2009. There was some evidence of reduced abundance of host-seeking *An. funestus s.l.* at households with livestock in 2007, but no difference was evident in other years (Table 3).

**Table 2:** **Mean abundance of malaria vectors caught per trap (per night) using different trapping methods during the study period (95% confidence intervals are given in brackets): CDC = CDC light trap used indoors, RB = an outdoor resting box, RC = a resting catch inside a house, RCA = a resting catch inside a cattle shed**

	**Households without livestock**			**Households with livestock**			
***An gambiae s.l.***	**CDC**	**RB**	**RC**	**CDC**	**RB**	**RC**	**RCA**
2007	0.136 (0.12-0.82)	0.01 (0–0.013)	0.05 (0.01-0.19)	0.55 (0.02-0.18)	0.02 (0.01-0.06)	0.13 (0.03-0.49)	3.94 (0.50-31.37)
2008	5.05 (2.72-9.37)	0.03(0.01-0.09)	0.04 (0.03-0.64)	8.04 (3.58-18.06)	0.07 (0.020.19)	0.15 (0.03-0.64)	1.04 (0.18-6.00)
2009	33.25 (11.34-95.77)	0.06 (0.04-0.18)	0.30 (0.06-1.36)	18.56 (8.26-41.72)	0.17 (0.08-0.17)	0.35 (0.14-0.90)	1.44 (0.23-8.99)
All years	2.02 (0.79-5.13)	0.03 (0.01-0.10)	0.13 (0.05-0.37)	2.18 (1.20-3.95)	0.06 (0.04-0.11)	0.07 (0.02-0.24)	1.30 (0.36-4.78)
***An. funestus***	**CDC**	**RB**	**RC**	**CDC**	**RB**	**RC**	**RCA**
2007	1.00 (0.41-2.43)	0.03 (0.01-0.09)	0.04 (0.01-0.21)	0.06 (0.02-0.17)	0.04 (0.02-0.08)	0.81 (0.01-0.21)	**
2008	0.69 (0.24-1.96)	0.002 (0–0.01)	0.08 (0.02-0.37)	0.68 (0.37-1.27)	0.01 (0.001-0.03)	0.03 (0.01-0.08)	**
2009	1.45 (0.51-4.17)	0.04 (0.02-0.09)	0.06 (0.02-0.20)	1.46 (0.79-2.71)	0.03 (0.01-0.08)	0.15 (0.05-0.47)	**
All years	0.87 (0.54-1.40)	0.02 (0.01-0.05)	0.07 (0.02-0.24)	0.75 (0.45-1.22)	0.01 (0.004-0.02)	0.06 (0.03-0.14)	0.32 (0.01-1.23)

**indicates where data were insufficient for estimation.

Estimates are given for each study year, and for the total over all years (where year was fit as a random effect).

**Table 3:** **Statistical significance of the impact of household livestock ownership on the abundance of indoor host seeking mosquitoes and malaria vector species composition across the three years of the study**

**Trait**	**Species**	**2007**		**2008**		**2009**	
		**Dev**	**P**	**Dev**	**P**	**Dev**	**P**
***Mean abundance***							
CDC light trap indoor	*An. gambiae s.l*	2.16	0.14	1.30	0.26	2.49	0.11
	*An. funestus s.l.*	4.67	0.03	1.41	0.26	1.37	0.24
***Species Composition***	CDC light trap	8.33	<0.01	55.25	<0.001	0.15	0.70
	Outdoor resting	1.18	0.28	4.30	0.04	8.54	<0.01
	Indoor resting	1.12	0.29	0.65	0.42	3.39	0.07

The abundance of host seeking mosquitoes (per night) was measured by CDC light traps placed indoors. Species composition refers to the proportion of *An. arabiensis* within the *An. gambiae s.l*. species complex. “Dev” = Deviance, and P values are for the significance of the statistical comparison between households with and without livestock.

**Figure 2:**
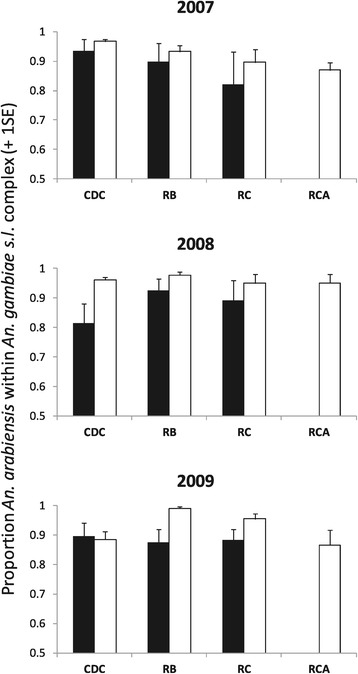
**Proportion of** ***Anopheles arabiensis*** **within the** ***Anopheles gambiae s.l.*** **species complex caught at households with (white bars) and without livestock (black bars) in different study years.** Trapping methods used were CDC = CDC light traps indoors, RB = outdoor resting boxes, RC = resting catches made inside houses and RCA = resting catches made inside livestock sheds.

In both resting and host-seeking collections, *An. arabiensis* was the most abundant member of the *An. gambiae s.l.* complex (Figure 2). By 2009, almost no *An. gambiae s.s.* were collected, confirming the near elimination of this species throughout the study area (Figure 2). In 2007 and 2008, *An. arabiensis* formed a slightly higher, statistically significant proportion of indoor-biting *An. gambiae s.l.* at households with livestock than without (Figure 2, Table 3), but there was no difference in 2009 (χ_1_^2^ = 0.15, p = 0.70). Similar, moderate but statistically significant increases in the proportion of *An. arabiensis* within outdoor resting collections were found at households with livestock in 2008 (χ_1_^2^ = 4.30, p = 0.04) and 2009 (χ_1_^2^ = 8.54, p < 0.01), but absent in 2007 (χ_1_^2^ = 1.18, p = 0.28). The proportion of *An. arabiensis* in indoor resting collections was unrelated to livestock in any study year (Figure 2, Table 3).

### Livestock and mosquito vector resting and feeding behaviour

As expected, substantially fewer mosquito vectors were captured in resting collections than in host seeking collections (Additional file 2). The number of *An. gambiae s.l.* and *An. funestus s.l.* caught in all resting collections (outdoor resting boxes, inside houses, inside cattle sheds) was equivalent to 6-8% and 3-14% of the total caught in host seeking collections respectively. Outdoor resting boxes were very effective in sampling mosquitoes, with the total number of *An. gambiae s.l.* caught resting outdoors being similar or higher to that caught inside houses (Additional file 2). However perhaps unsurprisingly on the basis of their differing surface area, the mean number of *An. gambiae s.l.* and *An. funestus s.l.* caught per collection was higher inside a house than in a single resting box (Table 3). The pattern of mosquito resting behavior varied notably between houses with and without livestock. Where cattle were present, more *An. gambiae s.l.* were found resting inside animals sheds than inside houses or outdoor resting boxes (Table 3). Where cattle were absent, most *An. gambiae s.l.* were found resting inside houses.

The number of *An. gambiae s.l.* and *An. funestus s.l.* found resting inside houses was generally very low (on average <0.5 per collection, Table 2), and had no in association with livestock (Table 2, p > 0.05 in all years). The abundance of *An. gambiae s.l.* found resting in outdoor resting boxes was significantly higher at households with livestock than without (Deviance = 5.76, p = 0.02, Table 2), but did not vary between years (Deviance =3.54, p = 0.17). In contrast the abundance of *An. funestus s.l.* in outdoor resting boxes varied between years (Deviance =1,408.5, p < 0.001, Table 2) but not with livestock availability (p < 0.05 all years).

The proportion of mosquitoes that were blood fed on capture varied between resting habitats and in relation to household livestock status. Overall, a higher proportion of mosquitoes were found blood fed in cattle sheds (65-80%), followed by inside houses (44–70 %|) and outdoor resting boxes (11-71%,). The proportion of mosquito vectors found blood fed was consistently higher at houses with than without livestock (Additional file 3). Of the 1,209 *An. gambiae s.l.* and 126 *An. funestus* were processed for blood meal identification, ~80% were identified as containing the blood of at least one of five assayed host species (human, cattle, goat, dog, and chicken). Generally, mosquitoes tested positive for only one type of host blood, but 3.9% tested positive for multiple host species (Additional file 4). Of these 41 mixed feeds, 39 were from households with livestock. The distribution of mixed feeds among vectors species was as follows: *An. arabiensis*: 38 (13 human and cattle, seven human and dog, 15 cattle and dog, one each cattle and goat, goat and dog, dog and chicken), *An. gambiae s.s.* two (all human and cattle) and *An. funestus s.l.* one (human and dog).

**Figure 3:**
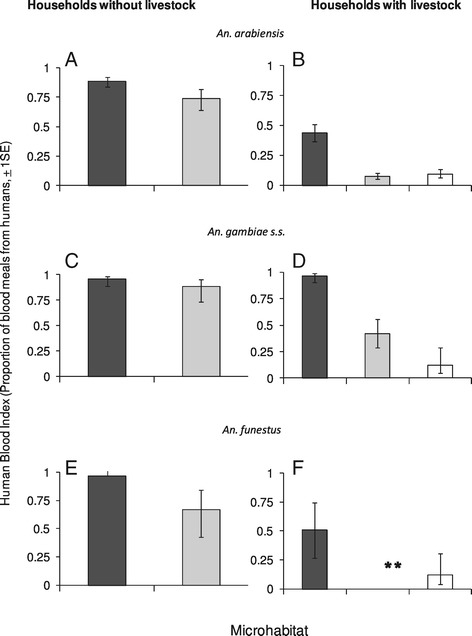
**The human blood index (HBI) of the three malaria vectors collected from different habitats at households with and without livestock in the Kilombero Valley.** Data pooled over all years of study (2007–09). Black bars are for HBI inside houses, grey for outdoor resting box, and white for cattle sheds. Error bars represent one standard error. Note: **indicates there were no blood-fed *An. funestus* collected inside houses at households with livestock.

The HBI of indoor-resting *An. arabiensis* (χ_1_^2^ = 42.93, p = <0.001, Figure 3A and B) and *An. funestus s.l.* (χ_1_^2^ = 28.44, p < 0.001, Figure 3E and F) was significantly lower at households with livestock than without, but no differences were observed in the HBI of *An. gambiae s.s.* (χ_1_^2^ = 0.01, p = 0.91, Figure 3C and D). The HBI of mosquitoes resting outside was significantly lower at households with livestock than without (*An. arabiensis*: χ_1_^2^ = 59.83, p < 0.001, Figure 3A and B: *An. gambiae s.s.*:χ_1_^2^ = 5.33, p = 0.02, Figure 3C and D). At households with livestock, the

HBI of vectors resting in cattle sheds was significantly lower than inside houses, but similar to that of mosquitoes resting outdoors (Figure 3B& 3D). At households with livestock, only a small number of blood-fed *An. funestus s.l.* (n = 11) were found resting outdoors and none of them had fed on humans (cattle n = 10 and goat n = 1). The HBI of *An. funestus s.l.* collected inside houses was significantly higher than in cattle sheds (χ_1_^2^ = 9.55, p = 0.01, Figure 3F).

### Livestock and human exposure to infected mosquito bites

A total of 2,537 *An. arabiensis*, 755 *An. gambiae s.s*, 857 *An. funestus s.l.,* and 8,755 pooled *An. gambiae s.l*. samples were tested for sporozoite infection status. Analysis of pools of *An. gambiae s.l.* that were not identified to species level (expected to be predominantly *An. arabiensis,* Figure 2) indicated that there was significant variation in sporozoite rates between study villages (χ_9_^2^ = 33.28, p < 0.001). Sporozoite rates in *An. gambiae s.l.* were predicted to be 0.33 and 0.10%, at households without and with livestock respectively (Table 4), but this difference was not statistically significant after controlling for random variation between villages (χ_1_^2^ = 2.15, p = 0.14). However, when background village-level variation was excluded from analysis and replaced by the ‘minimum distance to nearest household with livestock’ as an alternative proxy measure of spatial clustering, a significant association between household livestock ownership and *An. gambiae s.l.* sporozoite rates was detected (χ_1_^2^ = 4.62, p = 0.03, Table 4). Neither the year of collection (z-value = −0.667, p = 0.51) nor the minimum distance to another household with livestock (z-value =0.71, p = 0.48) were significantly associated with *An. gambiae s.l.* sporozoite rates in this analysis.

**Table 4:** **Sporozoite rates in malaria vectors that were tested in groups of unspeciated pools (within** ***Anopheles gambiae s.l.*** **complex), and within a subsample that were individually identified to species level**

**Vector species**	**Livestock status**	**Number tested**	**Percent infected (%)**	**95% CI (%)**
**Samples tested in pools**				
*An. gambiae s.l.*	Absent	5478	0.40	0.13-0.94
	Present	3277	0.10	0.008-0.39
**Individually tested specimens**				
*An. arabiensis*	NA	2537	0.39	0.20-0.78
*An. funestus*	NA	857	1.10	0.54-2.19
*An. gambiae s.s.*	NA	755	0.81	0.44-1.93

Samples sizes were only sufficiently large within the *An. gambiae s.l.* dataset to test for an impact of livestock presence. For the subsample of vectors whose species was individually confirmed, data were pooled over all years and household livestock types (thus household livestock defined as NA).

Analysis of sporozoite data from the subsample that were individually identified to species level (within *An. gambiae s.l.*) indicated that sporozoite rates varied significantly between vector species (χ_1_^2^ = 6.40, p = 0.04). Sporozoite rates were higher in *An. funestus s.l.* than *An. arabiensis* (z = 2.43, p = 0.002, Table 4), with *An. gambiae s.s*. being intermediary and not statistically different from either *An. arabiensis* or *An. funestus s.l.* (p > 0.10 in both cases).

## Discussion

In this study, the overall abundance of malaria vectors in both host seeking and resting collections was not consistently different between households with or without livestock. The abundance of mosquito vectors found host-seeking indoors and resting outdoors was slightly lower at households with cattle in only one of three study years. In other years there was no detectable difference. However, livestock ownership was associated with differences in malaria vector species composition, resting site usage and feeding behaviour. Over most years, *An. arabiensis* constituted a significantly higher proportion (5-15% more) of the indoor biting and outdoor resting *An. gambiae s.l.* population at households with cattle. Additionally, at households where cattle were present, significantly more vectors were found resting inside cattle sheds than inside houses or outdoor resting boxes. Further, the human blood index of

*An. arabiensis* and *An. funestus s.l.* was approximately 50% lower at households with livestock than without (inside houses and outdoor resting boxes respectively). These results confirm that the local presence of alternative host species such as cattle can significantly alter the habitat and host use of mosquito vectors at the household level.

Whilst the impact of cattle on mosquito vector behaviour was pronounced, the potential for these ecological effects to influence human malaria exposure risk was unclear. Malaria infection rates in *An. gambiae s.l.* collected from households with livestock tended to be lower than at those without livestock. However, the statistical significance of this effect depended on how background spatial variation in mosquito infection rates was controlled for. When village-level variation in mosquito sporozoite rates was incorporated into analysis, the impact of household livestock ownership was not significant. However, when village-level effects were removed and replaced by another proxy of spatial clustering, the nearest distance to another house (within the dataset) where livestock were kept, the difference in *An. gambiae s.l* sporozoite rates between

households with and without livestock achieved statistical significance. It was significantly higher at households without livestock than with.

The contrasting predictions obtained from different statistical models are deliberately presented here to highlight that no single unambiguous interpretation of these results is yet possible, and that further investigation to disentangle potentially confounding effects is required. At least two alternative explanations could account for the observed pattern. The first is that the reduced sporozoite rates found in *An. gambiae s.l.* is an indirect consequence of livestock keepers being more likely to live in villages where malaria transmission was lower; either by chance or due to co-occurring environmental conditions such as more open grassland, nearer distances to the river, etc., which could influence risk. Another potential explanation is that ‘village’ is too large or imprecise a measure over which to assume transmission is heterogeneous. The villages in this study area were not always discrete units with clear spatial separation between them. Some villages were immediately adjacent to each other whilst others covered relatively large areas with two or more population clusters within them. Recent evidence suggests that malaria exposure risk can vary significantly over distances of a few hundred metres in response to local environmental factors [57], thus there could have been significant heterogeneity in malaria transmission within these study villages that washed out finer-scale impacts of livestock at the household level. Finally, the tendency for lower sporozoite rates at households with livestock may be due to the higher proportion of *An. arabiensis* within the *An. gambiae s.l.* in these settings. Sporozoite rates were moderately lower in *An. arabiensis* than in *An. gambiae s.s.,* thus variation in the relative proportion of these two species within the vector community could influence the total exposure risk arising from *An. gambiae s.l.* This could provide an explanation for the observed variation in *An. gambiae s.l.* sporozoite rates, but does not help resolve whether it is likely to have a significant epidemiological impact. Further study investigating the contribution of environmental variation over multiple spatial scales to both these entomological indicators and clinical risk factors is required to definitively resolve the impact of cattle on exposure risk.

In this study, the most pronounced impact of livestock was a reduction in the human blood index of malaria vectors. This help to support the lower sporozoite rates observed at households with livestock. The higher the human-vector contact the higher the risk of malaria transmission [58]. However, the magnitude of the changes in HBI varied between vector species. At household with livestock, about 90% of non human blood index was from cattle, (Additional file 4). Whilst the HBI of *An. arabiensis* and *An. funestus s.l* was ~50% at households with livestock, *An. gambiae s.s.* was relatively unaffected. The consistently high human blood index of *An. gambiae s.s.* is not surprising in light of its well documented highly anthropophilic behaviour [20]. However, the sizeable reduction in the HBI of *An. funestus* was unexpected given this species is typically thought to be highly anthropophilic [20,59]. A possible explanation is that mosquitoes identified as *An. funestus s.l.* in this study included morphological cryptic species, which have more diverse behaviours. *Anopheles funestus s.l.* is a species complex consisting of both the type species (*An. funestus s.s.*) and 7 morphologically indistinguishable subspecies [60]. Of these, *An. funestus s.s.* was assumed to be the only member of the species complex present within the Kilombero Valley at the time of study as resources for molecular confirmation were not available. More recently, Lwetoijera *et al.* have confirmed that several members of this species complex are present in this area including *An. funestus s.s, Anopheles rivulorum*, *Anopheles leesoni* and *Anopheles parensis* [61]. Of these, *An. funestus s.s.* predominates by 98%. The presence of *An. rivulorum* which is highly zoophilic and is known to be associated with cattle [62] may account for the observed reduction in the HBI of *An. funestus s.l*. at households with cattle, or it can also mean that *An. funestus s.s.* did feed on livestock as well. A further study needs to be done to clear this observation.

The abundance of mosquito vectors collected by different sampling methods also raises the possibility of human exposure to mosquito bites was overestimated in this study. Between ten and twenty times more vectors were sampled by CDC light traps than in all resting collections combined. Although clearly more efficient for sampling, the number of vectors captured in CDC light traps may not accurately reflect the proportion that would succeed in feeding. In our study, the abundance of blood fed mosquitoes found resting indoors was very low (on average <0.5 mosquito per collection), whereas 3–4 times more found in resting catches inside cattle sheds. This may indicate that few mosquitoes who attempt to feed indoors are successful due to the presence of bed nets, with most leaving the house to seek blood elsewhere (possibly in cattle sheds). Under such a scenario, CDC light traps might have overestimated actual exposure rates in the presence of bed net use. Further investigations involving detailed study of house entry and exit behaviors under varying scenarios of bed net usage and cattle presence would be useful to test this possibility.

Whilst this study yielded no clear evidence of a protective effect of cattle on exposure to malaria vectors, the possibility of a detrimental, zoopotentiative effect was refuted. Neither the abundance nor sporozoite rates of indoor biting vectors were higher at households with livestock.

It has been hypothesized that keeping cattle could increase malaria risk by attracting more mosquitoes to nearby houses, providing an additional source of blood to fuel mosquito reproduction, and create more larval habitats (through the puddles their footprints create, etc.) [63]. This phenomenon has been observed in Ethiopia and Pakistan where the density of human-biting vectors increased in association with livestock [16,64]. However, these studies were conducted in communities where livestock were kept either inside human dwellings [16], or where people slept outside close to livestock [64]. In the Kilombero Valley, residents generally sleep indoors at night, with livestock being situated in separate cattle sheds that are an average of ≤25 m away. The separation of human and animal dwellings on this scale appears to be sufficiently large to avoid a zoopotentiation effect.

Differences in mosquito vector ecological and epidemiological factors described may be the cumulative impact not only of the presence of livestock, but of variation in socioeconomic and housing conditions that could be correlated with livestock keeping. For example, several household factors such as roof type, the presence of open eaves, window screens and ITN usage are significantly related to the abundance of malaria vectors that are find indoors at households within the Kilombero Valley [65,66]. Additionally, these factors are associated with wealth both in this part of Tanzania [67], and other parts of sub-Saharan Africa [68,69]. Thus any systematic variation in house type, bed net usage, and socioeconomic status between households with and without livestock here could confound our ability to identify the specific impact of cattle. Unfortunately it has not possible to collect contemporary data on these associated household factors within the scope of the current study, so we cannot rule this out as a possibility. We note anecdotally however, that no systematic differences in house construction between households with and without livestock were obvious in this study. Almost all houses in this area have open eaves (>90%, Mnyone *et al.,* unpublished data) and households spanning the range of very low (generally thatched roof and walls, no window screens) to moderate income (bricked walls, aluminium roofs, screened windows) were evident in both livestock classes. Bed nets were observed in almost every household visited, although the insecticidal property could not be ascertained. Additionally, variation in mosquito numbers between households may also have been influenced not only by local households features, but the proximity and density of hosts (human and cattle) at neighbouring households. Time and logistic constraints meant that it was not possible to simultaneously map the distribution of people and cattle at all surrounding households, and include this as additional explanatory variables in our analysis. To fully resolve the direct impact of cattle on malaria risk, we encourage further more detailed studies in which associated demographic and socioeconomic factors from both focal and neighbouring households are taken into consideration.

Analysis of mosquito resting site use presented here was based on comparison of the abundance of vectors found inside individual houses *versus* individual outdoor resting boxes. Generally these abundances were similar. However, when the total number of mosquito vectors captured inside a house *versus* all outdoor resting boxes (four to eight per site) onsite was summed, significantly more individuals were caught inside than outside. This indicates that if resting collections were made only from inside houses, as is typical in many vector surveillance studies, at least half of the local resting vector population (those resting outdoors) would be missed. By failing to monitor what can clearly be a significantly sized outdoor resting population, conventional indoor-based surveillance methods risk misrepresenting vector ecology, and missing opportunities to identify settings in which vector control could be significantly strengthened by targeting mosquitoes outside houses.

A limitation of the present study was that it only estimated exposure rate in terms of the number of infectious bites that people would be expected to receive when they were indoors between 18.00 and 06.00 hours. Given that *An. arabiensis* is exophilic [70], it is possible that outdoor biting rates and associated exposure risk is higher at households with livestock [15]. Further work to simultaneously quantify outdoor and indoor exposure risk at households with cattle is required to resolve this. However, a number of studies, including others from the Kilombero Valley [47,71] have shown that the biting activity of malaria vectors mainly occurs between 22:00 pm −06:00 am, a period when most people are asleep indoors [72]. Consequently, assessment of mosquito biting indoors is a relevant index of the majority of human exposure.

These results add to a growing body of research that suggests the potential effectiveness of zooprophylaxis will vary with ecological context. For example, a previous study in West Africa found no evidence that cattle could provide a zooprophylactic effect in reducing exposure or disease risk [17,21]. The dominant vector species in this study was the highly anthropophilic *An. gambiae s.s.* [21], whose innate host preference may render it less susceptible to a zooprophylaxis approach. In contrast, other studies conducted in areas of Kenya and Zambia where *An. arabiensis* is dominant found a significant reduction of malaria prevalence in areas where livestock were kept [31,33,34]. This variability highlights the need for detailed study of vector ecology and behaviour to identify settings in which combining relatively simple household-level interventions such as extending insecticide coverage to cattle and their holding facilities [63,73,74] with existing frontline measures (e.g., LLINs and IRS) could yield substantial improvements in malaria vector control.

## Additional files

Additional file 1:
**Figure A is the typical house of majority of livestock keeping households, B typical cattle shed.** C shows setting of resting box and D shows collection of mosquitoes from a resting box by aspiration. Additional file 2:
**Summary of the number of vectors collected in each year and the percent processed for species identification, blood meal source and sporozoite status.** NA = the samples were not processed for a named assay. Additional file 3:
**The blood feeding status of malaria vectors caught resting in different habitats (inside houses, outdoor resting boxes and cattle sheds) at houses with and without livestock present over all 3 study years.**
Additional file 4:
**Blood meal sources of the malaria vectors found in different resting habitats.**
