# Supplementary material for: The impact of livestock on the abundance, resting behaviour and sporozoite rate of malaria vectors in southern Tanzania
Source: Malar J. 2015 Jan 21;14:17. doi: 10.1186/s12936-014-0536-8 (PMC4311485; doi:10.1186/s12936-014-0536-8)

**Additional file 1 Figure A is the typical house of majority of livestock keeping households, B typical cattle shed. C shows setting of resting box and D shows collection of mosquitoes from a resting box by aspiration.**


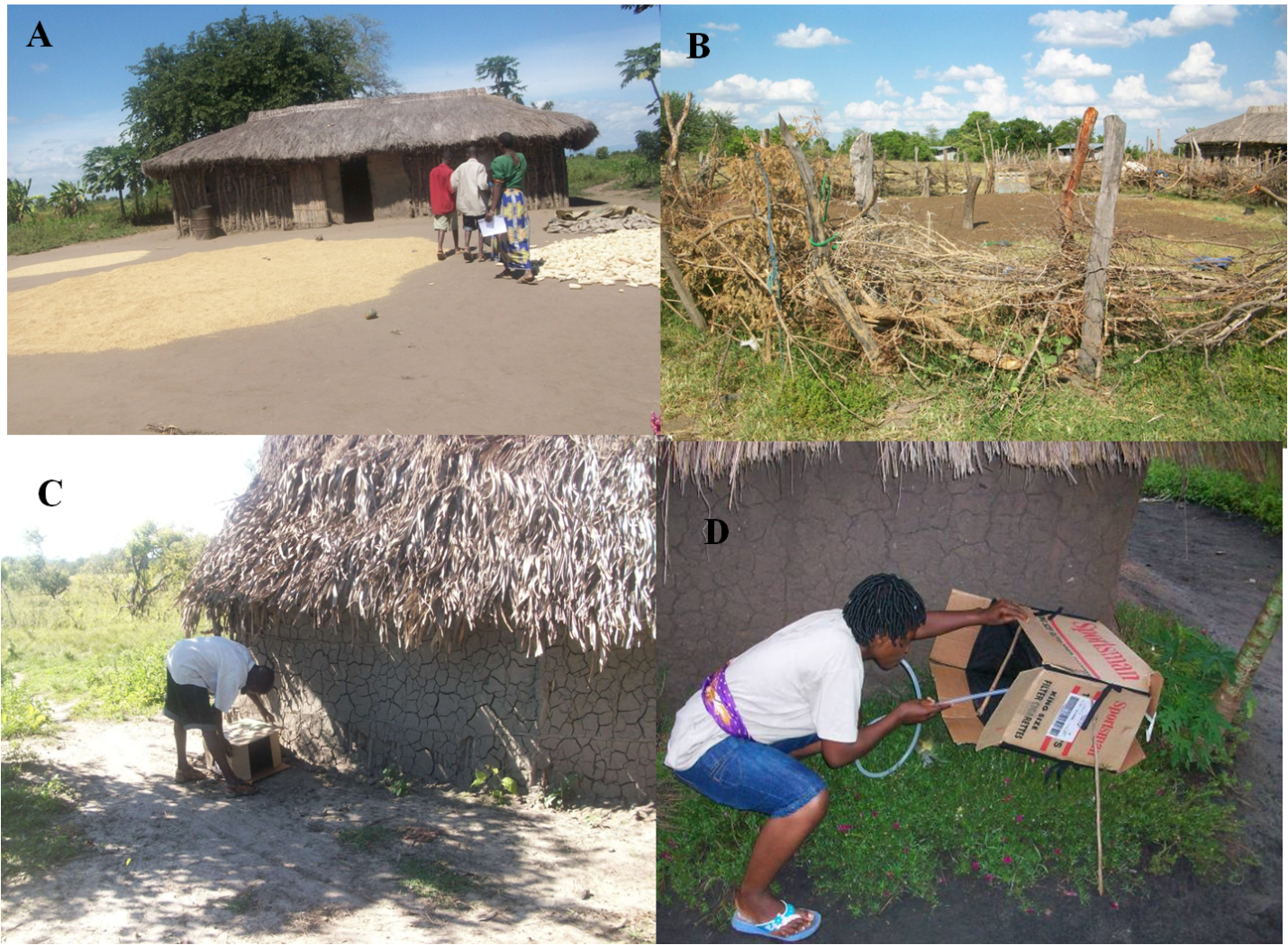

Supplement: Additional file 1: — Figure A is the typical house of majority of livestock keeping households, B typical cattle shed. C shows setting of resting box and D shows collection of mosquitoes from a resting box by aspiration. [file 12936_2014_536_MOESM1_ESM.docx]
