# Supplementary material for: The impact of livestock on the abundance, resting behaviour and sporozoite rate of malaria vectors in southern Tanzania
Source: Malar J. 2015 Jan 21;14:17. doi: 10.1186/s12936-014-0536-8 (PMC4311485; doi:10.1186/s12936-014-0536-8)
Supplement: Additional file 2: — Summary of the number of vectors collected in each year and the percent processed for species identification, blood meal source and sporozoite status. NA = the samples were not processed for a named assay. [file 12936_2014_536_MOESM2_ESM.docx]

**Additional file 2** **Summary of the number of vectors collected in each year and the percent processed for species identification, blood meal source and sporozoite status. NA = the samples were not processed for a named assay.**

| **Species** | **Trap type** | **Number collected** | **Species ID (%)** | **Blood meal ID (%)** | | **Sporozoite ID (%)** |
| --- | --- | --- | --- | --- | --- | --- |
|  | **2007** | | | | | |
|  | CDC LT | 1449 | 90 | NA | 89 | |
|  | Resting catch (indoor) | 245 | 91 | 52 | NA | |
|  | Resting catch (Animal shed) | 130 | 94 | 81 | NA | |
|  | Outdoor resting box | 542 | 78 | 40 | NA | |
|  | **2008** | | | | | |
| *An. gambiae s.l.* | CDC LT | 8659 | 30 | NA | | 99.8 |
|  | Resting catch (indoor) | 508 | 69 | 46 | | NA |
|  | Resting catch (Animal shed) | 257 | 82 | 64 | | NA |
|  | Outdoor resting box | 449 | 82.4 | 30 | | NA |
|  | **2009** | | | | | |
|  | CDC LT | 11,927 | 6 | NA | | 29 |
|  | Resting catch (indoor) | 953 | 33.4 | 23 | | NA |
|  | Resting catch (Animal shed) | 889 | 19 | 11 | | NA |
|  | Outdoor resting box | 798 | 65 | 15.5 | | NA |
|  | **TOTAL for all years** | **26806** |  |  | |  |
|  | **2007** | | | | | |
|  | CDC LT | 679 | NA | NA | | NA |
|  | Resting catch (indoor) | 298 | NA | NA | | NA |
|  | Resting catch (Animal shed) | 2 | NA | NA | | NA |
|  | Outdoor resting box | 76 | NA | NA | | NA |
|  | **2008** | | | | | |
| *An funestus s.l.* | CDC LT | 540 | NA | 8.04 | | 99.8 |
|  | Resting catch (indoor) | 60 | NA | 63.3 | | NA |
|  | Resting catch (Animal shed) | 16 | NA | 69.2 | | NA |
|  | Outdoor resting box | 31 | NA | 14.2 | | NA |
|  | **2009** | | | | | |
|  | CDC LT | 420 | NA | 4.3 | | 75 |
|  | Resting catch (indoor) | 116 | NA | 43.1 | | NA |
|  | Resting catch (Animal shed) | 227 | NA | 43.2 | | NA |
|  | Outdoor resting box | 122 | NA | 10.65 | | NA |
|  | **TOTAL for all years** | **2587** |  |  | |  |
