# Supplementary material for: The impact of livestock on the abundance, resting behaviour and sporozoite rate of malaria vectors in southern Tanzania
Source: Malar J. 2015 Jan 21;14:17. doi: 10.1186/s12936-014-0536-8 (PMC4311485; doi:10.1186/s12936-014-0536-8)
Supplement: Additional file 3: — The blood feeding status of malaria vectors caught resting in different habitats (inside houses, outdoor resting boxes and cattle sheds) at houses with and without livestock present over all 3 study years. [file 12936_2014_536_MOESM3_ESM.docx]

**Additional file 3:** The blood feeding status of malaria vectors caught resting in different

habitats (inside houses, outdoor resting boxes and cattle sheds) at houses with and without

livestock present over all 3 study years.

| **Method** | **Livestock**  **present** | **Total female collected** | **Total blood fed** | **% Blood fed** |
| --- | --- | --- | --- | --- |
| ***An. gambiae s.l.*** |  |  |  |  |
| Inside houses | No | 345 | 154 | 44.64 |
|  | Yes | 1042 | 733 | 70.09 |
| Outdoor boxes | No | 437 | 54 | 11.83 |
|  | Yes | 697 | 496 | 71.16 |
| Cattle sheds | Yes | 1201 | 968 | 80.60 |
| ***An. funestus*** |  |  |  |  |
| Inside houses | No | 262 | 157 | 59.92 |
|  | Yes | 108 | 70 | 64.81 |
| Outdoor boxes | No | 63 | 13 | 20.63 |
|  | Yes | 36 | 15 | 41.67 |
| Cattle sheds | Yes | 46 | 30 | 65.22 |
