# Supplementary material for: The impact of livestock on the abundance, resting behaviour and sporozoite rate of malaria vectors in southern Tanzania
Source: Malar J. 2015 Jan 21;14:17. doi: 10.1186/s12936-014-0536-8 (PMC4311485; doi:10.1186/s12936-014-0536-8)
Supplement: Additional file 4: — Blood meal sources of the malaria vectors found in different resting habitats. [file 12936_2014_536_MOESM4_ESM.docx]

**Additional file 4 Blood meal sources of the malaria vectors found in different resting habitats**

| Species | Livestock status | Resting site | Blood meal source | | | | | | | |
| --- | --- | --- | --- | --- | --- | --- | --- | --- | --- | --- |
|  |  |  | Human | Cow | Goat | Dog | Chicken | Mixed | Unknown | Total |
| *An arabiensis* | Present | Houses | 104 | 129 | 6 | 25 | 0 | 15 | 78 | 357 |
|  |  | Cattle sheds | 23 | 201 | 6 | 7 | 0 | 9 | 82 | 328 |
|  |  | Outdoors | 25 | 237 | 1 | 12 | 1 | 11 | 55 | 342 |
|  |  |  |  |  |  |  |  |  |  |  |
|  | Absent | Houses | 24 | 3 | 0 | 1 | 1 | 3 | 6 | 38 |
|  |  | Outdoors | 29 | 4 | 0 | 5 | 2 | 0 | 12 | 52 |
|  |  |  |  |  |  |  |  |  |  |  |
| *An gambiae s.s.* | Present | Houses | 23 | 0 | 0 | 1 | 0 | 0 | 2 | 26 |
|  |  | Cattle sheds | 1 | 7 | 0 | 0 | 0 | 0 | 3 | 11 |
|  |  | Outdoors | 3 | 4 | 0 | 1 | 0 | 2 | 7 | 17 |
|  |  |  |  |  |  |  |  |  |  |  |
|  | Absent | Houses | 22 | 0 | 0 | 1 | 0 | 0 | 6 | 29 |
|  |  | Outdoors | 8 | 0 | 0 | 1 | 1 | 0 | 2 | 12 |
|  |  |  |  |  |  |  |  |  |  |  |
| *An. funestus* | Present | Houses | 13 | 20 | 4 | 0 | 0 | 1 | 8 | 46 |
|  |  | Cattle sheds | 2 | 21 | 1 | 0 | 0 | 0 | 4 | 28 |
|  |  | Outdoors | 0 | 10 | 1 | 0 | 0 | 0 | 1 | 12 |
|  |  |  |  |  |  |  |  |  |  |  |
|  | Absent | Houses | 31 | 0 | 0 | 1 | 0 | 0 | 8 | 46 |
|  |  | Outdoors | 2 | 1 | 0 | 0 | 0 | 0 | 1 | 4 |
